# Impact of Transitioning Elective Orthopedic Surgeries to the Ambulatory Setting on Acute-Care Hospital Operations and Quality

**DOI:** 10.7759/cureus.100430

**Published:** 2025-12-30

**Authors:** Justin Turcotte, Andrea H Johnson, Madhulika C Nallani, Cathaleen D Ley, Paul J King, Sherry B Perkins

**Affiliations:** 1 Orthopedic and Surgical Research, Anne Arundel Medical Center, Annapolis, USA; 2 Orthopedics, Anne Arundel Medical Center, Annapolis, USA; 3 Administration, Anne Arundel Medical Center, Annapolis, USA; 4 Nursing Research, Anne Arundel Medical Center, Annapolis, USA; 5 Orthopedic Surgery, Anne Arundel Medical Center, Annapolis, USA

**Keywords:** ambulatory surgery center, case mix index, hospital-based care, nursing quality indicators, patient acuity, spine surgery, total joint arthroplasty

## Abstract

Introduction

Total joint arthroplasty (TJA) and spine surgeries are increasingly being performed in the ambulatory surgery center (ASC) setting. As high-volume elective surgeries shift out of the acute-care setting, the acuity and complexity of patients requiring hospital care have increased. The current study evaluates how the transition of TJA and spine procedures from the hospital to the ASC affected patient acuity and outcomes in a historically specialized hospital joint and spine unit (JSU).

Methods

A retrospective review of 12,067 patients receiving care at the JSU during calendar years 2018-2019 and 2022-2023 was performed. In 2021, our institution began performing TJA and spine procedures in a hospital-affiliated ASC. Calendar years 2020 and 2021 were excluded, as this period coincided with both the COVID-19 pandemic and the ASC ramp-up period. Bivariate analyses were performed using a pre-post intervention (i.e., ASC opening) design. Comparisons of patient characteristics and outcomes between the two periods were performed for all patients, patients undergoing total joint or spine surgery, and patients not undergoing total joint or spine surgery (i.e., other surgical or medical admissions to the unit). Operational measures, quality indicators, and staffing measures were compared between time periods for the entire unit.

Results

Compared to pre-ASC patients, those receiving care in the JSU post-ASC were older and had higher BMIs on average (both p<0.001). Furthermore, the post-ASC population was more diverse, with a greater percentage of Black (17.7% vs. 14.0%, p<0.001) and Medicaid patients (1.7% vs. 0.6%, p<0.001). Post-ASC, patients presented with a higher comorbidity burden as demonstrated by a higher Charlson Comorbidity Index (CCI) score, severity of illness, and risk of mortality scores (all p<0.001). Post-ASC, joint/spine surgery patients accounted for 71.0% of patients, compared to 90.7% pre-ASC (p<0.001). In contrast, ED arrivals increased from 5.3% to 24.2% of JSU patients post-ASC (p<0.001).

The average hospital length of stay (LOS) increased (pre-ASC 1.6 vs. post-ASC 2.6 days, p<0.001), and rates of 0/1 day LOS decreased while rates of 2+ day LOS increased (all p<0.001) post-ASC. In addition, rates of skilled nursing facility discharge and 30-day readmissions increased (both p<0.001), but 30-day ED return rates remained stable post-ASC. These trends were largely driven by the outcomes of non-total joint/spine surgery patients.

Post-ASC, the average daily census increased from 13.10 to 16.75 patients (p<0.001). CCI-adjusted nursing hours per patient day decreased significantly (pre-ASC 12.4 vs. post-ASC 11.0, p<0.001). Finally, overall unit turnover increased from 27% to 59% post-ASC.

Conclusion

This study highlights the changing patient landscape of a specialized orthopedic unit that has seen a growth in medically complex cases amidst the opening of a hospital-affiliated ASC. While these changes cannot be considered directly caused by the ASC opening, further evaluation of the impact of the migration of orthopedic surgeries to ASCs is warranted to confirm or refute the findings from this retrospective single-center study. These findings highlight the challenges of caring for a more racially and socioeconomically diverse population; hospitals should continue to refine strategies for delivering high-quality, equitable care as the demographics of patients treated in the acute-care setting evolve.

## Introduction

Total joint arthroplasty (TJA) and spine surgery are historically among the most commonly performed procedures in the acute-care hospital setting [1,2]. With the increased adoption of enhanced recovery after surgery (ERAS) protocols and shifts in insurance coverage policies, both TJA and spine surgeries are increasingly being performed as hospital outpatient procedures or in the ambulatory surgery center (ASC) setting, a trend that is expected to continue in the coming years [3-7].

As high-volume elective surgeries shift out of the acute-care setting, the acuity and complexity of patients requiring hospital care in the United States have increased, as evidenced by a 5% increase in the case mix index (CMI) and 10% increase in the inpatient average length of stay (LOS) from 2019 to 2023 [7]. Future projections suggest hospital case mix will continue to shift, as orthopedic and spine inpatient admissions are expected to decrease by 7% by 2033, offset by a 4%-7% growth in medicine, cardiovascular, and neuroscience services [7]. In response to these changes in patient population, hospitals will require unit redesign and reallocation of resources, each of which requires significant implementation time. To date, the majority of studies related to patient acuity and nursing care have focused on the variability in acuity across units and institutions, and the resulting implications for staffing levels, safety indicators, and patient outcomes [8-11]. A paucity of evidence evaluating the effects of shifts in care delivery models on nursing operations and patient outcomes, and the resulting changes in patient populations, exists.

The current study aims to fill this gap by evaluating the research question: how did the transition of TJA and spine procedures from the hospital to the ASC setting affect patient acuity and outcomes in a historically specialized hospital joint and spine unit (JSU)? To answer this question, we evaluated patient demographics, clinical characteristics (including multiple measures of comorbidity burden), social vulnerability and case mix before and after the opening of a hospital-affiliated ASC. Furthermore, we assessed patient outcomes such as LOS, discharge disposition, and 30-day emergency department (ED) returns or readmissions, as well as nursing quality indicators, staffing patterns, and turnover rates at the unit before and after the ASC opening. We hypothesized that the migration of cases to the ASC resulted in increased patient acuity in the unit and that operational efficiency and quality indicators were adversely affected. Although the study describes a single institution’s experience, the findings may be applicable across organizations and other units facing rapid changes in patient case mix. Our institution’s use of a dedicated JSU is not unique, nor are the challenges presented by a continually changing healthcare environment. While the specific changes that occur may vary across institutions, this study provides a framework for evaluating their impact that may be applied broadly.

## Materials and methods

Study population

A retrospective review of patients receiving care at the JSU at Luminis Health Anne Arundel Medical Center during calendar years 2018-2019 and 2022-2023 was performed. Luminis Health Anne Arundel Medical Center is a single, 377-bed acute care hospital serving an urban, suburban, and rural population in Maryland. The institution has approximately 94,000 emergency department visits and 23,000 inpatient medical admissions annually. In 2021, our institution began performing TJA and spine procedures in a de novo hospital-affiliated ASC. Calendar years 2020 and 2021 were excluded from the analysis, as this period coincided with both the COVID-19 pandemic and the ASC ramp-up period after the center’s opening in 2021. The potential effects of the exclusion of these years on internal validity are described further in the Limitations section. No additional exclusion criteria were applied.

Study design

All analyses were performed using a pre-post intervention (i.e., ASC opening) design. Calendar years 2018-2019 were designated as the pre-ASC opening period and 2022-2023 as the post-ASC opening period. Comparisons of patient characteristics and outcomes between the two periods were performed for all patients, patients undergoing total joint or spine surgery, and patients not undergoing total joint or spine surgery (i.e., other surgical or medical admissions to the unit). Operational measures, quality indicators, and staffing measures were compared between time periods for the entire unit. All data were extracted from the hospital electronic medical record (EMR) and nursing quality indicator systems using the analytics and reporting suite; no manual chart review was performed.

Independent variables

Patient characteristics were divided into three sub-categories: demographics, clinical characteristics, and social vulnerability. Demographics assessed included age, body mass index (kg/m^2^), race (classified as Black/African American or all other races; all other races were 95% White, 3.5% Other race, 1.5% unknown/declined to answer), marital status (classified as married/life partner or unmarried/no life partner), and insurance status (classified as Medicaid or Other). Clinical characteristics included whether a patient arrived through the ED and multiple measures of comorbidities. The primary comorbidity measure used was the Charlson Comorbidity Index (CCI), as this can be calculated for all patients regardless of admission status (inpatient, outpatient, extended recovery/observation). Additional comorbidity measures assessed included CMI (inpatients only), Severity of Illness (SOI, inpatients only), risk of mortality (inpatients only), and American Society of Anesthesiologists score (surgical patients only). Social vulnerability was assessed using the Centers for Disease Control and Prevention/Agency for Toxic Substances and Disease Registry Social Vulnerability Index (CDC/ATSDR SVI). Overall SVI and the four dimensions of the SVI were assessed based on patients’ zip-code of residence. Scores range from 0 to 1 and represent the percentile ranking of the patient’s zip-code in comparison to the United States, with higher scores indicating greater levels of vulnerability. Given that the SVI is measured at the zip-code level, it does not capture within-area disparities in social vulnerability, or the specific socioeconomic status of individual patients, which may differ from that of their zip-code of residence. A description of the methodologies used to calculate these indices is presented in Table 1.

**Table 1:** Summary of included risk adjustment, comorbidity, and socioeconomic indices MS-DRG, Medicare Severity Diagnosis-Related Group; ICD-10, International Classification of Diseases, 10th Revision; APR-DRG, All Patient Refined DRG

Index/Score Name	Description
Charlson Comorbidity Index	Weighted index that predicts the risk of death within one year of hospitalization for patients with specific comorbid conditions [12]. As of 2024, there are 19 conditions listed in the index. Each condition has a weight assigned to it determined by a Cox proportional hazards model, and the higher the number, the greater the risk of dying within one year [13,14]. For each comorbid condition a patient has, a weight is assigned and then summed to determine the cumulative risk of death for a patient within one year.
Case mix index (CMI)	Determined by calculating the average of the relative weights of the MS-DRG for each inpatient treated at a hospital over a specific period; as of 2024, there are a total of 766 MS-DRGs [15,16]. The weight of each patient hospitalization is assigned based on patient attributes, including principal diagnosis, specific secondary diagnoses, procedures, sex, and discharge status from ICD-10 codes [17]. The CMI reflects the diversity, clinical complexity, and resource needs of all the patients in the hospital, with a higher CMI indicating a more complex case load [15].
Severity of Illness (SOI) score	A severity score is assigned to a patient at discharge, based on seven dimensions: stage of the principal diagnosis, complication of the principal condition, concurrent interacting conditions that affect the hospital course, dependency on hospital staff, extent of nonoperating room life support procedures, rate of response to therapy/rate of recovery, and impairment remaining after therapy for the acute aspect of the hospitalization [18]. Each dimension is rated on a scale of 1 (least severity) to 4 (highest severity) as per criteria, and an overall severity score is rated on a scale of 1 to 4 [18]. SOI values are assigned APR-DRGs as a reimbursement modifier, using the 3M grouper (3M Health Information Systems, Salt Lake City, USA) [19]. However, the measure has limited utility in cross-category comparison, as similar SOI levels across different APR-DRGs do not indicate the same level of disease burden [19].
Risk of mortality (ROM)	ROM is assigned to APR-DRGs using the 3M grouper based upon age, sex, diagnoses, and procedures coded to an inpatient hospital encounter. The ROM ranges from 1 (low) to 4 (extreme) [20]. SOI and ROM are independent classifications; a high SOI with low ROM (or vice versa) is possible depending on the specific diagnoses and procedures associated with a hospitalization [21].
American Society of Anesthesiologists (ASA) score	A score by the ASA physical status classification system to determine the health of a person before undergoing a surgery that requires anesthesia. The qualitative assessment uses a scale of I (normal, healthy patient) to VI (brain-dead patient), and is determined by the anesthesiologist immediately prior to surgery to ensure accuracy [22].
Social Vulnerability Index (SVI)	The CDC SVI was used to quantify socioeconomic disadvantage. Patients were assigned SVI scores based on the average SVI score of the census tracts within their zip-code of residence [23]. The SVI scoring methodology is built upon 16 variables calculated at the census-tract level. The 16 variables are used to calculate percentile rankings for each census tract across four themes. To calculate the overall SVI, the percentile rankings across the four themes are summed for each census tract. These are then ordered, and the overall percentile ranking is calculated [24].
Socioeconomic status	Includes data on percent of population below 150% of the poverty level, percent of population unemployed civilians, percent of households spending >30% of annual income on housing, percent of population with no high school diploma, percent of population without health insurance [23].
Household characteristics	Includes data on percent of population aged 65 and older, percent of population aged 17 and younger, percent of civilians with a disability, percent of single-parent households, percent with English language proficiency [23].
Racial and ethnic minority status	Includes data on percent of minority population (defined as Hispanic/Latino, Black/African American, American Indian and Alaska Native, Native Hawaiian and other Pacific Islander, two or more races, other race) [23].
Housing type and transportation	Includes data on percent in multi-unit housing, percent in mobile homes, percent in crowded conditions, percent of households with no vehicle available, percent of population residing in group quarters [23].

Outcomes

Commonly used measures of hospital quality were assessed at the patient level. These included inpatient versus outpatient status, LOS and the percentage of 0, 1, and 2+ day LOS, rates of discharge to skilled nursing facilities (SNFs), 30-day ED returns, and 30-day readmissions. Operational measures assessed at the unit level included average daily census (measured as 24-hour census), intakes (the number of patients arriving at the unit during a calendar day), discharges (number of patients discharged from the unit during a calendar day), and churn percentage (intakes + discharges/daily census). The average daily census was not adjusted for unit closures or changes in bed capacity. Quality indicators assessed at the unit level included three measures from the National Database of Nursing Quality Indicators (NDNQI): injury falls per 1000 patient days, catheter-associated urinary tract infections (CAUTIs) per 1000 catheter days, and percent of surveyed patients with hospital-associated pressure injuries (HAPIs) ≥ Stage 2 [25]. Levels of unit staffing were measured as nursing hours per patient day (NHPPD). Total NHPPD, including all direct caregivers (i.e., registered nurses, or RN; licensed practical nurse, or LPN; and techs) and RN-only NHPPD, were evaluated. CCI-adjusted NHPPD was calculated using the following formula: CCI-Adjusted NHPPD = Unadjusted NHPPD/(Time period average CCI/Total study period average CCI). Turnover rates for all JSU employees, bedside RNs, and non-bedside RNs (all other jobs on the unit) were calculated as the number of employees terminated (voluntary or involuntary) divided by the average employee count during the study periods.

Data analysis

Bivariate analyses were used to compare patient characteristics and outcomes between the pre-ASC opening and post-ASC opening periods. Two-sided independent samples t-tests were performed to compare continuous measures, and chi-square tests were performed to compare categorical measures. The nursing quality indicators were compared using Mann-Whitney U tests, as these measures are calculated on a monthly basis leading to an insufficient number of observations and non-normal distribution of data for performance of parametric statistics. All data were analyzed as available; no imputation was performed for missing variables. Data analysis was intentionally performed without adjustment for confounding factors to examine the changes in patient characteristics and outcomes as experienced over the study period. All statistical analysis was performed in IBM SPSS Statistics, v.26 (IBM, Armonk, NY). Statistical significance was assessed at the p<0.05 level.

Ethical approval

This study was deemed exempt by the institutional review board (WCG IRB) as it was a retrospective review of existing medical records, and a HIPAA waiver was granted.

## Results

Compared to pre-ASC patients, those receiving care at the JSU after the opening of the ASC were older and had higher BMIs on average (both p<0.001). Furthermore, the post-ASC population was more diverse, with a greater percentage of Black/African American patients (n=981 (17.7%) vs. 913 (14.0%), p<0.001), Medicaid patients (n=94 (1.7%) vs. 38 (0.6%), p<0.001), and patients without a spouse or life partner (n=2,258 (40.8%) vs. 2,082 (31.9%), p<0.001). Overall, SVI scores were similar between periods, but greater vulnerability in the dimension of racial and ethnic minority status (p=0.028) and less vulnerability in the household characteristics dimension (p=0.037) were observed after the ASC opening. During the post-ASC period, patients presented with a higher comorbidity burden as demonstrated by higher CCI scores (all patients), severity of illness and risk of mortality scores (inpatients only), and ASA scores (surgical patients only) (all p<0.001). Notably, CMI scores for inpatients showed the inverse trend (pre-ASC 2.6 ± 1.1 vs. post-ASC 2.5 ± 1.4, p=0.006). Despite this deviation in CMI, these measures generally indicate that a higher acuity patient population was treated at the unit post-ASC opening. Significant differences in case mix were also observed between periods. After the opening of the ASC, joint/spine surgery patients accounted for 71.0% (n=3,930) treated at the unit, compared to 90.7% (n=2,082) during the pre-ASC period (p<0.001). In contrast, ED arrivals increased from 5.3% (n=344) to 24.2% (n=1,342) for JSU patients post-ASC (p<0.001) (Table 2).

**Table 2:** Joint and spine unit patient characteristics: all patients p-values <0.05 are presented in bold; all data are presented as n (%) or mean ± SD. Continuous data are compared using a two-sided independent samples t-test; categorical data are compared using the chi-square test. ASC, ambulatory surgery center; BMI, body mass index; CCI, Charlson Comorbidity Index; IP, inpatient; ASA, American Society of Anesthesiologists; ED, emergency department

Patient Characteristic	Pre-ASC Opening (n=6,532)	Post-ASC Opening (n=5,535)	Test Statistic	p-Value
Age (years)	65.7 ± 11.2	68.7 ± 13.0	t = -13.375	<0.001
Age >75 years	1,197 (18.3)	1,745 (31.5)	χ^2^ = 283.226	<0.001
BMI (kg/m^2^)	30.7 ± 6.0	30.2 ± 6.3	t = 4.045	<0.001
BMI> 35 kg/m^2^	1,499 (22.9)	1,221 (22.1)	χ^2^ = 1.356	0.253
Black/African American	913 (14.0)	981 (17.7)	χ^2^ = 31.778	<0.001
Not married or no life partner	2,082 (31.9)	2,258 (40.8)	χ^2^ = 103.538	<0.001
Medicaid	38 (0.6)	94 (1.7)	χ^2^ = 34.523	<0.001
Total joint/spine surgery	5,923 (90.7)	3,930 (71.0)	χ^2^ = 774.106	<0.001
ED arrivals	344 (5.3)	1,342 (24.2)	χ^2^ = 897.900	<0.001
CCI score (all patients)	2.7 ± 1.5	2.9 ± 1.7	t = -8.983	<0.001
Case mix index (IP only)	2.6 ± 1.1	2.5 ± 1.4	t = 2.734	0.006
Severity of illness (IP only)	1.7 ± 0.6	1.9 ± 0.8	t = -8.536	<0.001
Risk of mortality (IP only)	1.3 ± 0.5	1.5 ± 0.8	t = -13.675	<0.001
ASA score (surgical patients only)	2.4 ± 0.6	2.6 ± 0.6	t = -13.774	<0.001
Overall social vulnerability	0.27 ± 0.18	0.27 ± 0.17	t = 0.433	0.665
Socioeconomic status	0.26 ± 0.17	0.26 ± 0.16	t = 0.732	0.464
Household characteristics	0.37 ± 0.14	0.36 ± 0.14	t = 2.091	0.037
Racial and ethnic minority status	0.49 ± 0.23	0.50 ± 0.24	t = -2.202	0.028
Household type and transportation	0.31 ± 0.18	0.31 ± 0.17	t = -0.272	0.785

The characteristics of the pre- and post-ASC populations stratified by joint/spine surgery and other patient types are presented in Tables 3-4.

**Table 3:** Joint and spine unit patient characteristics by patient type: joint and spine surgery patients p-values <0.05 are presented in bold; all data are presented as n (%) or mean ± SD. Continuous data are compared using a two-sided independent samples t-test; categorical data are compared using the chi-square test ASC, ambulatory surgery center; BMI, body mass index; CCI, Charlson Comorbidity Index; IP, inpatient; ASA, American Society of Anesthesiologists; ED, emergency department

Patient Characteristic	Pre-ASC Opening (n=5,923)	Post-ASC Opening (n=3,930)	Test Statistic	p-Value
Age (years)	65.4 ± 10.8	68.9 ± 10.6	t = -16.043	<0.001
Age >75 years	1,009 (17.0)	1,122 (28.5)	χ^2^ = 184.785	<0.001
BMI (kg/m^2^)	30.8 ± 5.8	30.8 ± 5.7	t = -0.020	0.984
BMI> 35 kg/m^2^	1405 (23.7)	946 (24.1)	χ^2^ = 0.159	0.708
Black/African American	862 (14.6)	698 (17.8)	χ^2^ = 18.238	<0.001
Not married or no life partner	1,858 (31.4)	1,448 (36.8)	χ^2^ = 31.770	<0.001
Medicaid	31 (0.5)	51 (1.3)	χ^2^ = 17.163	<0.001
Total joint surgery	4,439 (74.9)	3,001 (76.4)	χ^2^ = 2.562	0.115
Spine surgery	1,484 (25.1)	929 (23.6)	χ^2^ = 2.562	0.115
ED arrivals	138 (2.3)	135 (3.4)	χ^2^ = 10.712	0.001
CCI score (all patients)	2.6 ± 1.5	2.7 ± 1.4	t = -4.377	<0.001
Case mix index (IP only)	2.6 ± 1.1	3.3 ± 1.5	t = -13.109	<0.001
Severity of illness (IP only)	1.7 ± 0.6	1.7 ± 0.7	t = -0.980	0.327
Risk of mortality (IP only)	1.3 ± 0.5	1.3 ± 0.6	t = -2.866	0.004
ASA score	2.4 ± 0.5	2.5 ± 0.5	t = -11.055	<0.001
Overall social vulnerability	0.27 ± 0.18	0.27 ± 0.18	t = 0.303	0.762
Socioeconomic status	0.26 ± 0.17	0.26 ± 0.17	t = 0.333	0.739
Household characteristics	0.37 ± 0.14	0.36 ± 0.14	t = 2.001	0.045
Racial and ethnic minority status	0.49 ± 0.24	0.50 ± 0.24	t = -1.081	0.282
Household type and transportation	0.31 ± 0.18	0.31 ± 0.18	t = -0.387	0.698

**Table 4:** Joint and spine unit patient characteristics by patient type: non-total joint and spine surgery patients p-values <0.05 are presented in bold; all data are presented as n (%) or mean ± SD. Continuous data are compared using a two-sided independent samples t-test; categorical data are compared using the chi-square test. ASC, ambulatory surgery center; BMI, body mass index; CCI, Charlson Comorbidity Index; IP, inpatient; ASA, American Society of Anesthesiologists; ED, emergency department

Patient Characteristic	Pre-ASC Opening (n=609)	Post-ASC Opening (n=1,605)	Test Statistic	p-Value
Age (years)	68.2 ± 14.2	67.9 ±17.6	t = 0.358	0.720
Age >75 years	188 (30.9)	623 (38.8)	χ^2^ = 12.008	<0.001
BMI (kg/m^2^)	29.1 ± 6.9	28.7 ± 7.6	t = 1.205	0.229
BMI> 35 kg/m^2^	94 (15.4)	275 (17.1)	χ^2^ = 0.917	0.371
Black/African American	51 (8.4)	283 (17.6)	χ^2^ = 29.539	<0.001
Not married or no life partner	224 (36.8)	810 (50.5)	χ^2^ = 33.220	<0.001
Medicaid	7 (1.1)	43 (2.7)	χ^2^ = 4.680	0.045
Orthopedic surgery	495 (81.3)	748 (46.6)	χ^2^ = 215.600	<0.001
Other surgery	56 (9.2)	182 (11.3)	χ^2^ = 2.116	0.168
Medical admission only	58 (9.5)	675 (42.1)	χ^2^ = 210.979	<0.001
ED arrivals	206 (33.8)	1,207 (75.2)	χ^2^ = 327.344	<0.001
CCI score (all patients)	3.2 ± 1.8	3.4 ± 2.3	t = -2.419	0.016
Case mix index (IP only)	2.3 ± 0.8	1.8 ± 0.9	t = 11.731	<0.001
Severity of illness (IP only)	1.7 ± 0.7	2.0 ± 0.8	t = -6.935	<0.001
Risk of mortality (IP only)	1.4 ± 0.6	1.7 ± 0.8	t = -8.376	<0.001
ASA score (surgical patients only)	2.5 ± 0.6	2.7 ± 0.6	t = -6.014	<0.001
Overall social vulnerability	0.27 ± 0.17	0.27 ± 0.16	t = -0.299	0.767
Socioeconomic status	0.26 ± 0.16	0.25 ± 0.16	t = 0.284	0.777
Household characteristics	0.36 ± 0.14	0.36 ± 0.13	t = 0.103	0.920
Racial and ethnic minority status	0.46 ± 0.21	0.50 ± 0.23	t = -3.629	<0.001
Household type and transportation	0.31 ± 0.18	0.31 ± 0.17	t = 0.455	0.656

The trends in the overall population were largely similar in both subgroups, with some notable exceptions. In joint/spine surgery patients, ED arrivals accounted for 2.3% (n=138) and 3.4% (n=135) of patients during the pre- and post-ASC periods, respectively (p=0.001). In non-total joint/spine surgery patients, the increase in ED arrivals was more pronounced, accounting for 33.8% (n=206) and 75.2% (n=1,207) of patients during the pre- and post-ASC periods, respectively (p<0.001). Furthermore, a shift in case mix of non-joint/spine surgery patients was noted as the percentage of other orthopedic surgery patients declined from 81.3% (n=495) to 46.6% (n=748) between periods (p<0.001), while medical admissions increased from 9.5% (n=58) to 42.1% (n=675) (p<0.001). A comparison of hospital outcomes between periods is presented in Tables 5-7.

**Table 5:** Joint and spine unit patient outcomes: all patients p-values <0.05 are presented in bold; all data are presented as n (%) or mean ± SD. Continuous data are compared using a two-sided independent samples t-test; categorical data are compared using the chi-square test. ASC, ambulatory surgery center; SNF, skilled nursing facility; ED, emergency department; LOS, length of stay

Outcome	Pre-ASC Opening (n=6,532)		Post-ASC Opening (n=5,535)	Test Statistic	p-Value
Inpatient		4,299 (65.8)	2,296 (41.5)	χ^2^ = 715.801	<0.001
LOS (days)		1.6 ±1.4	2.6 ± 3.6	t = -18.227	<0.001
0 day LOS		354 (5.4)	189 (3.4)	χ^2^ = 28.023	<0.001
1 day LOS		4,052 (62.0)	2,987 (54.0)	χ^2^ = 80.228	<0.001
2+ day LOS		2,126 (32.5)	2,359 (42.6)	χ^2^ = 130.157	<0.001
Discharge to SNF		659 (10.1)	83 (15.0)	χ^2^ = 67.140	<0.001
30-day ED return		296 (4.5)	278 (5.0)	χ^2^ = 1.595	0.223
30-day readmission		180 (2.8)	214 (3.9)	χ^2^ = 11.701	<0.001

**Table 6:** Joint and spine unit patient outcomes: total joint and spine surgery patients p-values <0.05 are presented in bold; all data are presented as n (%) or mean ± SD. Continuous data are compared using a two-sided independent samples t-test; categorical data are compared using the chi-square test. ASC, ambulatory surgery center; SNF, skilled nursing facility; ED, emergency department; LOS, length of stay

Outcome	Pre-ASC Opening (n=5,923)		Post-ASC Opening (n=3,930)	Test Statistic	p-Value
Inpatient	3,792 (64.0)		1,126 (28.7)	χ^2^ = 1182.231	<0.001
LOS, days	1.6 ± 1.3		1.8 ± 2.4	t = -5.109	<0.001
0 day LOS	325 (5.5)		184 (4.7)	χ^2^ = 3.126	0.085
1 day LOS	3,742 (63.2)		2,674 (68.0)	χ^2^ = 24.599	<0.001
2+ day LOS	1,856 (31.3)		1,072 (27.3)	χ^2^ = 18.628	<0.001
Discharge to SNF	507 (8.6)		286 (7.3)	χ^2^ = 5.251	0.024
30-day ED return	273 (4.6)		171 (4.4)	χ^2^ = 0.365	0.579
30-day readmission		151 (2.5)	78 (2.0)	χ^2^ = 3.318	0.080

**Table 7:** Joint and spine unit patient outcomes: non-total joint and spine surgery patients p-values <0.05 are presented in bold; all data are presented as n (%) or mean ± SD. Continuous data are compared using a two-sided independent samples t-test; categorical data are compared using the chi-square test. ASC, ambulatory surgery center; SNF, skilled nursing facility; ED, emergency department; LOS, length of stay

Outcome	Pre-ASC Opening (n=609)		Post-ASC Opening (n=1,605)	Test Statistic	p-Value
Inpatient	507 (83.3)		1,170 (72.9)	χ^2^ = 25.762	<0.001
LOS (days)	2.1 ± 1.8		4.4 ± 5.0	t = -16.089	<0.001
0 day LOS	29 (4.8)		5 (0.3)	χ^2^ = 57.827	<0.001
1 day LOS	310 (50.9)		313 (19.5)	χ^2^ = 215.285	<0.001
2+ day LOS	270 (44.3)		1,287 (80.2)	χ^2^ = 271.920	<0.001
Discharge to SNF	152 (25.0)		545 (34.0)	χ^2^ = 16.569	<0.001
30-day ED return	23 (3.8)		107 (6.7)	χ^2^ = 6.671	0.013
30-day readmission		29 (4.8)	136 (8.5)	χ^2^ = 8.814	0.004

Across the full population, a shift towards increased management of patients in outpatient status was observed during the post-ASC period. Furthermore, the average hospital LOS increased (pre-ASC 1.6 ± 1.4 vs. post-ASC 2.6 ± 3.6 days, p<0.001), and rates of 0 and 1 day LOS decreased while rates of 2+ day LOS increased (all p<0.001) during the post-ASC period. In addition, rates of SNF discharge and 30-day readmissions increased (both p<0.001), but 30-day ED return rates remained stable post-ASC opening. These trends appear to be largely driven by the outcomes of non-total joint/spine surgery patients in the unit. In this population, the average LOS increased from 2.1 ± 1.8 to 4.4 ± 5.0 days (p<0.001) with rates of 2+ day LOS increasing from 44.3% (n=270) to 80.2% (n=1,287) (p<0.001) between periods. Additionally, rates of SNF discharges (p<0.001), 30-day ED returns (p=0.013), and 30-day readmissions (p=0.004) all increased during the post-ASC period. In contrast, joint/spine surgery patient average LOS increased slightly from 1.6 ± 1.3 to 1.8 ± 2.4 days, but rates of 2+ day LOS declined from 31.3% (n=1,856) to 27.3% (n=1,072) post-ASC. In this subset of patients, rates of SNF discharge decreased from 8.6% (n=507) to 7.3% (n=286) (p=0.024), while 30-day ED return and readmission rates remained stable post-ASC opening.

After the opening of the ASC, the average daily census increased from 13.10 ± 5.79 to 16.75 ± 4.47 patients (p<0.001). However, churn rates decreased from 136.24 ± 70.42 to 89.42 ± 39.77% (p<0.001). No significant differences in nursing quality indicators, including fall, CAUTI, and HAPI rates, were observed between periods (Table 8).

**Table 8:** Joint and spine unit operational and quality indicators ^a^Data are presented as means ± SDs; a two-sided independent samples t-test was performed ^b^Data are presented as median (interquartile range); Mann-Whitney U test was performed. p-values <0.05 are presented in bold; Churn percentage = Intakes + Discharges/Daily census CAUTI, catheter-associated urinary tract infection; HAPI, hospital-associated pressure injury

Operational Measure	Pre-ASC Opening (n=6,532)	Post-ASC Opening (n=5,535)	Test Statistic	p-Value
Average daily volume^a^				
Census	13.10 ± 5.79	16.75 ± 4.47	t = -13.480	<0.001
Intakes	8.94 ± 7.18	7.57 ± 4.91	t = 4.261	<0.001
Discharges	8.95 ± 5.79	7.58 ± 4.42	t = 5.066	<0.001
% Churn	136.24 ± 70.42	89.42 ± 39.77	t = 15.541	<0.001
Quality indicators^b^				
Injury falls per 1000 patient days	0.41 (0.00-1.35)	0.00 (0.00-0.66)	U = 25.000	0.505
CAUTI per 1000 catheter days	0.00 (0.00-0.00)	0.00 (0.00-4.67)	U = 35.000	0.798
% of surveyed patients with HAPI ≥ Stage 2	0.00 (0.00-0.00)	0.00 (0.00-5.56)	U = 40.000	0.189

With regard to unit staffing, no differences in actual NHPPD (pre-ASC 11.8 ± 1.3 vs. post-ASC 11.5 ± 1.1, p=0.444) or RN/LPN-only NHPPD (pre-ASC 7.3 ± 0.8 vs. post-ASC 7.4 ± 0.8, p=0.741) were observed between periods. However, CCI-adjusted NHPPD rates decreased significantly both overall (pre-ASC 12.4 ± 1.5 vs. post-ASC 11.0 ± 1.3, p<0.001) and in RN/LPN only (pre-ASC 7.7 ± 0.9 vs. post-ASC 7.0 ± 1.0, p=0.021) (Table 9).

**Table 9:** Monthly unit staffing trends p-values <0.05 are presented in bold; all data are presented as means ± SDs. NHPPD, nursing hours per patient day; ASC, ambulatory surgery center; RN, registered nurse; CCI, Charlson Comorbidity Index; CCI-adjusted NHPPD = Unadjusted NHPPD/(Time period avg. CCI/Total study period avg. CCI)

Trend	Pre-ASC Opening	Post-ASC Opening	Test Statistic	p-Value
Actual NHPPD	11.8 ± 1.3	11.5 ± 1.1	t = 0.772	0.444
Actual NHPPD, RN only	7.3 ± 0.8	7.2 ± 0.8	t = 0.386	0.701
CCI-adjusted NHPPD	12.4 ± 1.4	11.0 ± 1.3	t = 3.541	<0.001
CCI-adjusted NHPPD, RN only	7.7 ± 0.9	6.9 ± 1.0	t = 3.006	0.004
Skill mix, % RN NHPPD	62.0 ± 1.7%	62.5 ± 2.8%	t = -0.835	0.409

**Figure 1:**
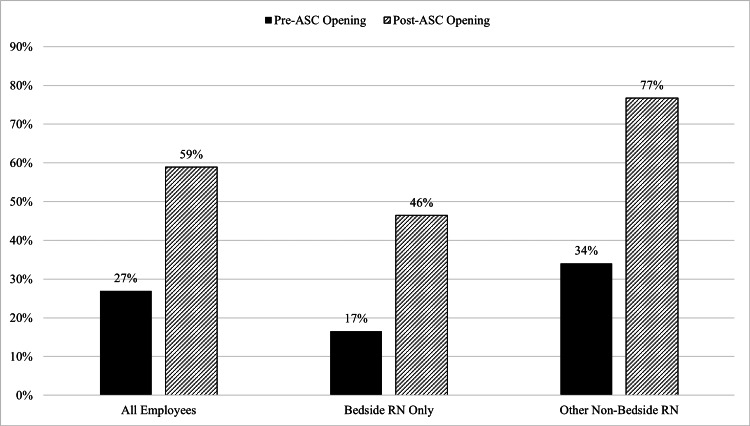
Total joint and spine unit (JSU) staff turnover rate All data are presented as % turnovers of staff categories. ASC, ambulatory surgery center; RN, registered nurse

Finally, overall unit turnover increased from 27% to 59%, bedside RN turnover increased from 17% to 46% and non-bedside RN (i.e., all other staff) turnover increased from 34% to 77% between the pre- and post-ASC periods (Figure 1).

## Discussion

At our institution, the opening of hospital-affiliated ASCs and the associated shift of elective TJA and spine surgeries out of the acute-care setting were associated with significant changes in the case mix, operations, and outcomes of patients receiving care at the JSU. The study demonstrates the transition of the JSU from a specialized orthopedic unit focused on throughput to a more generalized one, more closely resembling a medical/surgical floor serving a diverse patient population. After the opening of the ASC, patients treated in the unit were more medically complex across multiple indicators and more likely to present through the ED, and the average daily census increased. These changes were primarily due to the increased rate of non-joint/spine surgical patients placed on the JSU. While the outcomes of joint/spine surgery patients remained relatively stable, these new populations required longer LOS, and were more likely to discharge to SNFs and return to the hospital as ED arrivals or inpatients within 30 days of discharge. On an unadjusted basis, nursing hours per patient day remained stable over time, but were significantly lower when adjusting for comorbidities. In parallel with these changes in acuity, case mix, and workload, staff turnover increased substantially at the JSU during the post-ASC period.

While our study highlights the increased acuity and diversity (both in case mix and racial/socioeconomic status) of patients at the JSU after the opening of the ASC, its observational nature precludes our ability to identify the cause of this change. We hypothesize that this was most likely due to the hospital relaxing the unit’s joint and spine focus and “backfilling” it with the more diverse medical and surgical patient population. This theory is supported by the significant increases in medical admissions and ED arrivals to the unit during the post-ASC period. However, other systemic factors may have led to the changes observed. Prior studies have shown that Black patients, patients with public health insurance, and those residing in rural areas are less likely to undergo procedures in the ASC setting [26]. This disparity in access to ASC care may have influenced the increased proportion of Black/African American and Medicaid patients, as well as the differences in SVI scores observed after the ASC opening. Finally, it is possible that the increased acuity observed in the post-ASC period was influenced by the COVID-19 pandemic. Although this study excluded 2020-21, the residual effects of delays in care during this period were significant, particularly among patients with chronic conditions [27,28]. It is likely that a combination of these factors and their interaction influenced the results observed in this study, necessitating further investigation to isolate the effect of each on patient characteristics and outcomes in the acute-hospital setting.

The magnitude of change that occurred at the JSU following the shift of elective joint and spine procedures to the ASC is an obvious takeaway from the current study, but raises the question of whether the unit was adequately staffed to care for a more heterogeneous and medically complex patient population. At our institution, unit-staffing budgets are established based upon historical NHPPD and expected patient days without risk adjustment, and flexed based on actual volume. It is therefore unsurprising that we observed similar overall and RN-only NHPPD at the unit between periods, but decreased staffing ratios after adjusting for patient CCI. However, based on our data, we are unable to discern the reason for or potential confounding factors that contributed to this difference. It is possible that a variety of factors, including budget constraints, staff reallocation, or changes in hospital triage policies or patient throughput, contributed to the observed results. Multiple approaches to acuity-adjusted staffing have been described, including the use of case mix index, nursing intensity weight, and patient population-specific scales (e.g., infant acuity scale) as adjustment factors, along with proprietary tools [10,11,29]. While a comprehensive review of these alternatives is outside the scope of this work, each relies on the accuracy and rigor of documentation and coding to be valid. Interestingly, while we generally observed increasing acuity across most measures, the CMI reflected an inverse trend of decreased acuity during the post-ASC period, which may have been a result of coding or diagnosis-related group changes. However, our results suggest that real-time quantitative evaluation of the increasing acuity in the JSU may have enabled us to increase staffing to maintain the intended historical ratios. Based upon the existing literature demonstrating the positive association between increased nurse staffing and improved patient outcomes, it is possible that additional staffing may have mitigated some of the increases in length of stay, non-home discharges, and readmissions observed at the unit during the post-ASC period [30-32].

A second finding of this study was the significant increase in staff turnover that occurred during the post-ASC period. However, this must be considered in the context of the trend of increased nurse and clinical staff turnover nationwide that occurred over the study period. Most notably, during the COVID-19 pandemic, turnover of all hospital staff and RNs increased from 17.8% and 15.9%, respectively, in 2019 to 25.9% and 27.1% in 2021, according to a survey of 400 hospitals in 36 states [33]. Increased workloads, scarcity of resources, vulnerability to infection, and emotional distress have been identified as contributors to low levels of job satisfaction and burnout among nurses during the pandemic period [34-37]. At an average cost of over $56,000 per staff RN turnover, nursing shortages and increased labor costs are unsurprisingly among the top concerns of health system executives [33,38]. In this study, the turnover rate of 46% among RNs in the JSU during the post-ASC period far outpaced the national averages of approximately 21%-23% during the same period nationally, and other units at our institution [33]. Despite the external changes described, and the fact that one cannot infer causality from the observational nature of this study, the potential strain placed upon clinical staff caring for a more diverse and complex population cannot be dismissed as a potential contributor to the increased turnover observed. This strain may have also been amplified by the potential understaffing of the unit, previously described, as improving nurse staffing levels was identified as the top intervention for improving well-being reported by over >80% of 15,378 nurses surveyed at 60 U.S. Magnet-designated hospitals [39].

The trends observed in the current study suggest that additional interventions are needed to enable the delivery of high-quality, sustainable nursing care in the dynamic modern hospital environment. The American Association of Critical-Care Nurses (AACCN) and American Nurses Association (ANA) Nurse Staffing Taskforce Imperatives, Recommendations, and Actions guidelines provide actionable steps that may be taken to address the challenges faced by the JSU in this study, and others nationally [40]. Grouped into five imperatives, reform the work environment, innovate the models for care delivery, establish staffing standards that ensure quality care, improve regulatory efficiency, and value the unique contribution of nurses, the taskforce generated 16 recommendations for improving nurse staffing. While each of the recommendations holds value independently, we suggest the data presented in this study most directly highlight the need to “modernize care delivery models and ensure they are inclusive, evidence-informed, and technologically advanced.” Applied to our experience at the JSU, the implementation of innovative models for evaluating patient acuity and care complexity may enhance our ability to appropriately allocate the resources needed to reduce nurse workload and cognitive burden, potentially leading to improved patient outcomes and reduced staff turnover. As a tangible first step, we intend to implement the CCI-adjusted staffing model described to improve our resource allocation as patient mix and acuity continue to evolve. However, the adjustment of staffing patterns is only a first step towards optimizing the workforce to care for evolving patient populations. Continued evaluation of novel care delivery models, new technologies, and multidisciplinary collaboration is necessary to equip staff for these challenges in the ever-changing acute care environment.

Limitations

The results of this study must be considered in the context of its limitations. As a single-institution study, our findings may not be generalizable across hospitals and geographic regions. It is important to recognize the unique characteristics of this hospital that have shaped its surgical volumes, patient acuity, and staffing, and how that would significantly differ based on the geography and scope of services provided by each individual institution. Furthermore, the trends observed reflect the changes in a highly specialized joint and spine unit, specifically. While we assessed the implications of transitioning elective orthopedic surgical procedures to the ASC in this setting, further study is warranted to evaluate the effects of these shifts on overall hospital operations and of transitioning other procedure types out of the acute hospital setting. Additionally, as an observational study, there are a multitude of confounding factors besides the opening of the ASC that may have influenced our results, most notably, the aftermath of the COVID-19 pandemic. In alignment with national trends, it is likely that pandemic staffing burnout most likely exacerbated staff turnover and highly influenced the staffing trends observed. Furthermore, the exclusion of calendar years 2020-2021 may have introduced selection bias in the pre-post comparison, given the important shocks to hospital operations (i.e., COVID-19 and ASC ramp-up) that occurred during this period. Other unmeasured potential confounders, such as the specific conditions treated, changes in leadership and operational processes, and staff satisfaction, likely contributed to the observed results. Moreover, key operational details such as unit staffing structure and patient flow logistics were not evaluated and may have influenced the results. It is also possible that internal policy changes, such as ED triage and admission criteria, occurred over the study period and contributed to the results observed. Additionally, while we attempted to quantify the changes in racial and socioeconomic characteristics of patients treated at the unit, evaluation of the specific social determinants of health of each individual was not performed. In conducting our analyses, we purposefully refrained from controlling for the measurable changes in patient characteristics between study periods, to allow the impact of these differences to be reflected in our results as they occurred in reality. Therefore, there are a multitude of both measured and unmeasured changes during the pre- and post-ASC periods that likely influenced the observed results. Based on these limitations of the study design, one cannot infer that the relationship between the transition of joint and spine cases to the ASC setting and the observed changes in both patient mix and outcomes was causal in nature, and only an association may be inferred.

## Conclusions

This study highlights the changing patient landscape of a specialized orthopedic unit that has seen a growth in medically complex cases amidst the opening of a hospital-affiliated ambulatory surgery center. While these changes cannot be considered directly caused by the ASC opening, further evaluation of the impact of the migration of orthopedic surgeries to ASCs is warranted to confirm or refute the findings of this retrospective single-center study. Additionally, there are a multitude of confounding factors that may have contributed to the observed results. Further investigation isolating the effects of patient and operational changes, in addition to shifts in care settings using multicenter designs, is needed. As surgical procedures continue to transition towards outpatient sites of care, hospital leaders may consider how to effectively modify staffing allocations to keep pace with rapid changes in patient mix and acuity. Furthermore, the study highlights the challenges of caring for a more racially and socioeconomically diverse population; hospitals should continue to refine strategies for delivering high-quality, equitable care as the demographics of patients treated in the acute-care setting evolve.
